# Identification of multiple raisins by feature fusion combined with NIR spectroscopy

**DOI:** 10.1371/journal.pone.0268979

**Published:** 2022-07-14

**Authors:** Yajun Zhang, Yan Yang, Chong Ma, Liping Jiang

**Affiliations:** 1 College of Software, Xinjiang University, Urumqi, China; 2 College of Information Science and Engineering, Xinjiang University, Urumqi, China; 3 College of Information Engineering, Changji University, Changji, China; Accra Technical University, GHANA

## Abstract

Varieties of raisins are diverse, and different varieties have different nutritional properties and commercial value. In this paper, we propose a method to identify different varieties of raisins by combining near-infrared (NIR) spectroscopy and machine learning algorithms. The direct averaging of the spectra taken for each sample may reduce the experimental data and affect the extraction of spectral features, thus limiting the classification results, due to the different substances of grape skins and flesh. Therefore, this experiment proposes a method to fuse the spectral features of pulp and peel. In this experiment, principal component analysis (PCA) was used to extract baseline corrected features, and linear models of k-nearest neighbor (KNN) and linear discriminant analysis (LDA) and nonlinear models of back propagation (BP), support vector machine with genetic algorithm (GA-SVM), grid search-support vector machine (GS-SVM) and particle swarm optimization with support vector machine (PSO- SVM) coupling were used to classify. This paper compared the results of four experiments using only skin spectrum, only flesh spectrum, average spectrum of skin and flesh, and their spectral feature fusion. The experimental results showed that the accuracy and Macro-F1 score after spectral feature fusion were higher than the other three experiments, and GS-SVM had the highest accuracy and Macro-F1 score of 94.44%. The results showed that feature fusion can improve the performance of both linear and nonlinear models. This may provide a new strategy for acquiring spectral data and improving model performance in the future. The code is available at https://github.com/L-ain/Source.

## 1. Introduction

Raisins are rich in dietary fiber, vitamins, carotenoids and polyphenols [1]. Studies have found that consumption of raisins can not only regulate blood pressure, anti-inflammation and prevent cardiovascular diseases but also improve intestinal flora, protect nerves and improve spatial memory [2–4]. Grapes are mass-produced as crops, and raisins are also the raw materials of many foods. In 2017 only, the global production of raisins reached 1.22 million tons. Among them, the output of raisins in China accounted for 190,000 tons, ranking third [5]. In addition, grape varieties are very diverse and different varieties of raisins have different taste, nutritional properties and commercial value [6]. Currently, the ability to quickly and accurately identify different types of raisins is one of the main challenges facing quality ranking efforts and avoiding fraud.

In the existing studies, Mostafa et al. used raisins images combined with support vector machine (SVM) and linear discriminant analysis (LDA) to identify fifteen kinds of raisins, with the highest average accuracy of 69.78% [7]. Although their research has strong application significance, different classes of raisins are similar in color and shape, which increases the difficulty for images feature extraction and recognition. Zhao et al. used hyperspectral images combined with neural networks to identify eight kinds of raisins, with the highest accuracy of 82.22% [6]. In addition, hyperspectral images combined with principal component analysis (PCA) and radial basis function neural network were used to identify two kinds of raisins. Their research explored the feasibility of spectral signal for classification of raisins [8]. Other commonly used species identification methods include liquid chromatography-mass spectrometry, gas chromatography-mass spectrometry and DNA sequencing [9]. However, these methods not only need specific experimental sites and operators but also the experimental process is complicated and the detection cost is expensive. Compared with the above methods, the identification method of NIR is convenient, simple and economical. Therefore, this study proposed a method for rapid identification of different types of raisins by NIR spectroscopy combined with machine learning algorithms.

In addition, in the study of origin identification of almonds, it was found that the tegument was formed at the early growth stage, and the kernel was more affected by factors such as water deficiency in the external environment [10]. In the experiment, they compared the results of only using the NIR spectra of tegument and the average spectra of the tegument and kernel. The results showed that the classification accuracy was affected by only using spectral data of tegument [11]. Therefore, in the experiment of Zhao et al., they only recorded spectral data on the skin of raisins, which may be part of the reason why the model performance in the experiment was limited [6]. This also means that only recording the spectral data on the skin will ignore the molecular information of the flesh. To fully explore the molecular information contained in raisins, spectral data of skin and meat were collected separately in this study.

In the food identification of NIR spectra, the spectral data obtained from each sample is usually averaged directly, and then the processed data are used to train the model. Since mature grapes form a wax layer in skin, the flesh mainly contains carbohydrates and unsaturated fatty acids [12]. There are also differences in the content of polyphenols, lipids and carbohydrates between the skin and the flesh [13]. In addition, the content of aromatic compounds in raisins can be affected by drying promoter solutions, such as sodium hydroxide, citric acid, and potassium sulfate [16, 17]. If the spectral data of the skins and pulp are averaged directly, not only the data available for training the model will be reduced, but also the spectral features may be affected. Given these differences in material composition, the present study combined the spectral characteristics of the peel and pulp in our experiments and compared the results with three other experiments that used only the peel spectrum, only the pulp spectrum and its average spectrum.

This experiment used NIR spectroscopy combined with machine learning algorithms to identify three kinds of raisins: Hongxiangfei, Manaiti and Munage. Firstly, PCA was used to extract features from the spectra of skin and flesh, then merged the two types of features into a new feature matrix, and finally established k-nearest neighbor (KNN), LDA, BP(back propagation) and SVM (support vector machine) classification models. Compared with the four experimental results, the study demonstrates that morphological feature fusion can improve the model’s classification performance.

## 2. Materials and methods

### 2.1 Sample preparation

This experiment collected three kinds of raisins from Xinjiang, including Hongxiangfei, Manaiti and Munage. The harvest time of all samples was 2020. The aromatic compounds of raisins volatilize at high drying temperature [14, 15]. To avoid the influence of moisture content on the spectral data, the collected samples were stored in dry atmospheric environment for two weeks, and then each type of samples were sealed in a Ziplock bag for storage. Before starting to measure the near-infrared spectrum, take out the sample, flatten the surface of each raisin, and then record two spectra on the upper and lower surfaces respectively. After completion, cut from the middle with a knife to ensure that both sides of the incision are smooth, and then record a spectrum of the pulp on both sides.

### 2.2 Measurement of NIR spectra and preprocessing

A VERTEX 70 FT-IR spectrometer was used, and CO_2_ compensation was selected as the atmospheric compensation parameter. The scan range was 4,000–11,000 cm^-1^; the resolution was 8 cm^-1^; and the number of scans was 16; spectral measurement mode is diffuse reflectance; the NIR spectral dimension is 1814. Four skin spectra and two flesh spectra were recorded for each sample. Finally, the skin and flesh spectra of each sample were averaged, respectively. The average spectra of skin and flesh were to average the spectra of the skin and flesh of the same sample.

**Fig 1:**
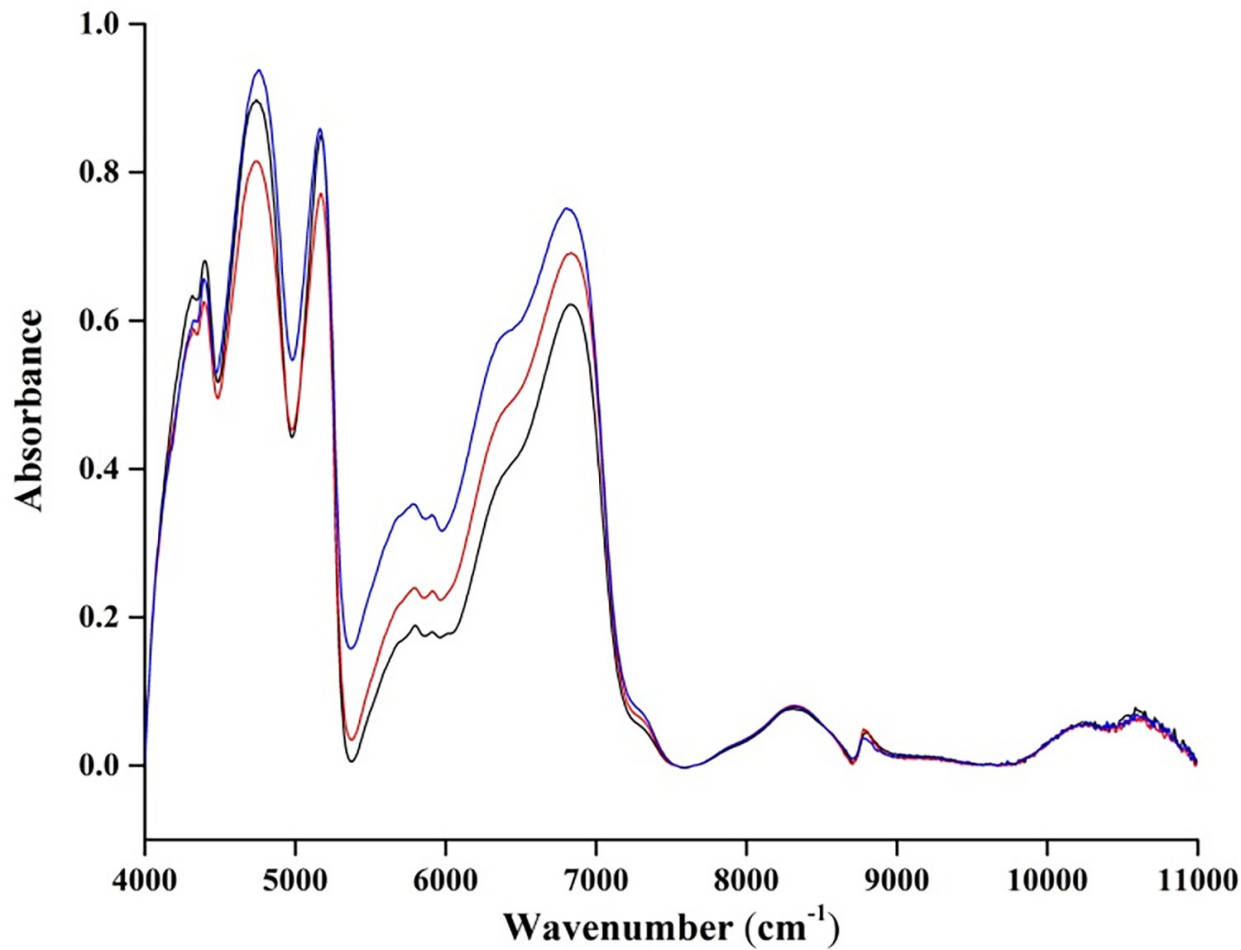
Arbitrary three baseline-corrected skin spectra.

**Fig 2:**
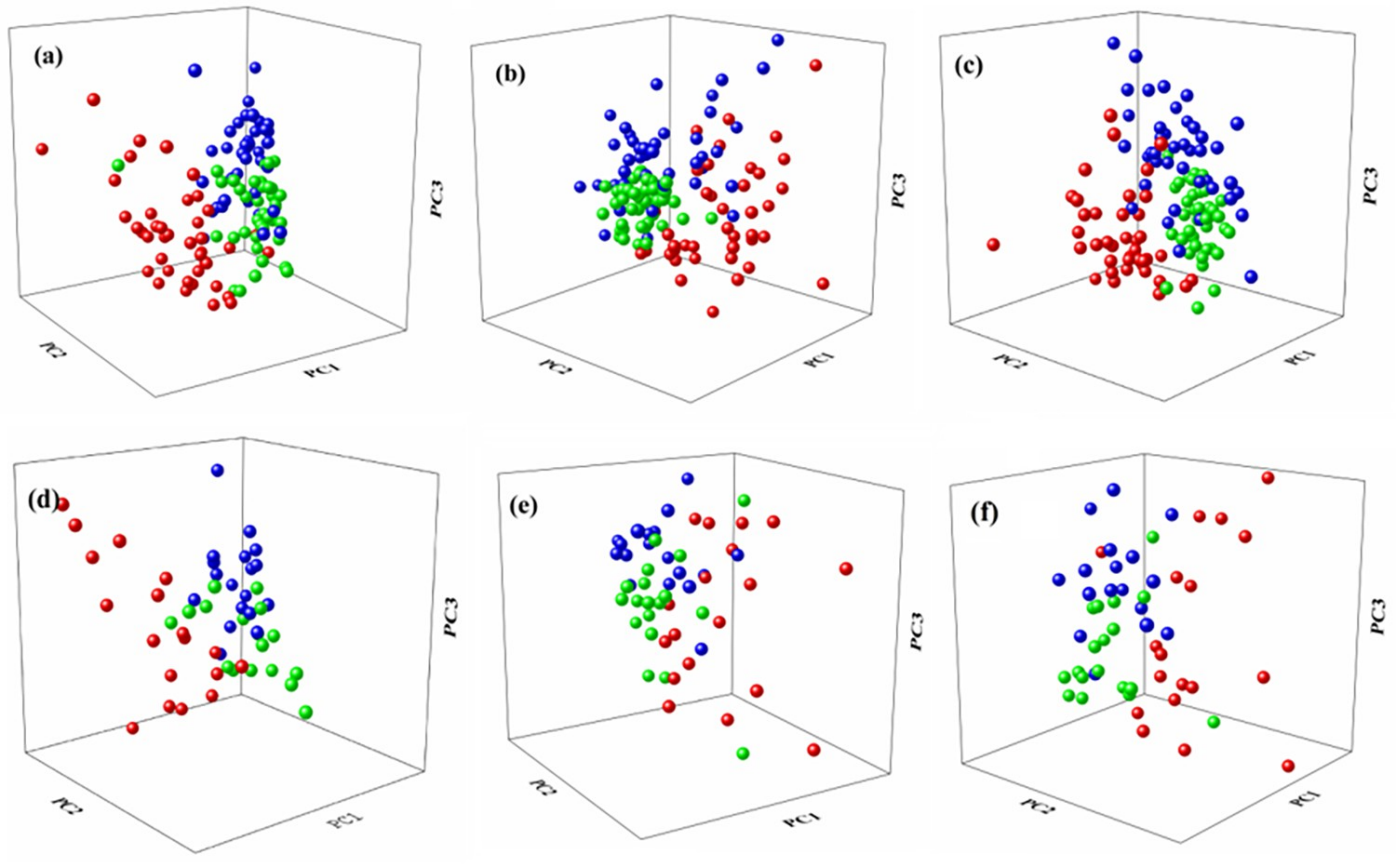
The first three PCs scatter plot in training set of **a** skin spectra, **b** flesh spectra, and **c** average spectra of them; the first three PCs scatter plot in test set of **d** skin spectra, **e** flesh spectra, and **f** average spectra of them. (The red balls represent samples of Hongxiangfei, the blue balls present samples of Manaiti and the green balls represent samples of Munage).

The raw spectra has high dimension and carries redundant information, which cannot be directly used as the training data of the models [16]. PCA is an unsupervised feature extraction method. It uses linear transformation to make features orthogonal to each other. The proportions of each principal component are shown in Table 1. In the experiments with peel, pulp and mean spectra, the principal components with 99% of the cumulative variance in the first four dimensions were selected in this study. To avoid the influence of electronic drift on spectra, rubberband algorithm in OPUS 7.8 software was used as the baselines of spectra correction and the baseline point value was set to 64. As shown in Fig 1, the corrected spectral data no longer have the problem of baseline drift. The processed data were randomly divided into training set and test set according to the ratio of 7:3. In the training set, there are 41 samples of Hongxiangfei and 42 samples of Manaiti and Munage. Meanwhile, the number of three classes of samples is 18 in the test set. In all experiments, the samples in the training set and test remained unchanged. PCA (MATLAB R2016b) was used for feature extraction, and then the processed data were used to train the models of KNN, LDA, BP and SVM (MATLAB R2016b). Ten-fold cross-validation was used for the models. Fig 2 is the first three-dimensional principal components (PCs) scatter plot of training set and test set.

**Table 1:** The proportion of each principal component in the cumulative variance.

	Skin	Flesh	Average
**PC1 (%)**	89.80	91.15	91.69
**PC2 (%)**	7.45	6.32	5.51
**PC3 (%)**	1.55	1.43	1.66
**PC4 (%)**	0.47	0.48	0.46

## 3. Results and comparison

### 3.1 Spectral analysis

**Fig 3:**
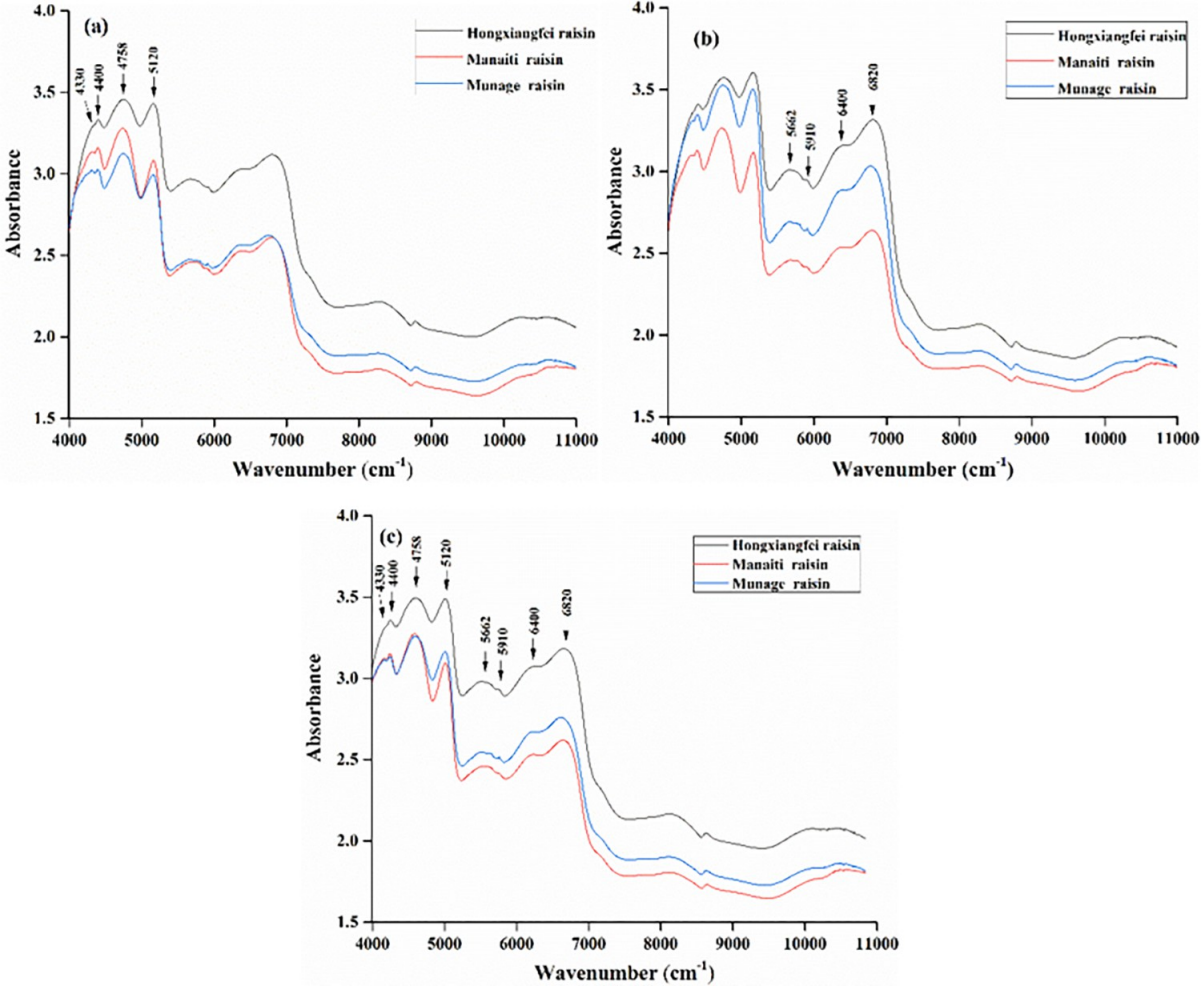
Average raw spectra of **a** skin, **b** flesh, and **c** both of them.

Fig 3(A)–3(C) are the average spectra of skin, flesh and both of them, respectively. Fig 3(A) shows that the difference in spectral characteristic peaks of skin is more obvious than that of flesh at 4330, 4440 and 4758 cm^-1^; in Fig 3(B), the difference of characteristic peaks of flesh spectra is more obvious than that of skin spectra at 5650, 5910, 6400 and 6820 cm^-1^. Table 2 shows the different infrared absorption bands corresponding to molecular information according to the above characteristic peaks and existing spectral studies [17–20].

**Table 2:** Peak positions and tentative assignments of the major NIR bands of raisins.

Wavenumber (cm^-1^)	Molecular information
4330, 4400	Phenols
4758	C-O stretching and O-H deformation
5120	C = O groups of the carbohydrates
5662	C-H of the flavone glycoside, fatty acids
5910	Amino acids
6400	Cellulose
6820	O-H of the flavonoids

Mature grapes form a wax layer in skin and the content of lipids [12], ketones and alcohols in wax layer were different [21]. Moreover, there were great differences in phenolic content of different grape skin [13]. Since the NIR spectral absorption band of phenols is 4300–4400 cm^-1^ [22], which explains that the absorption band of skin at 4330 and 4400 cm^-1^ is more significantly different than that of flesh.

The studies also found that the content of fatty acids, amino acids and volatile components in flesh of different grape varieties were also different [1, 23]. Moreover, the content of monosaccharides in different kinds of raisins was also very different [1]. Since the absorption bands of carboxylic acid and amino acid are 5814–6061 and 5920 cm^-1^ [22], respectively. Moreover, carboxylic acid is an important part of fatty acids. This is consistent with the characteristic peak of the flesh spectra at 5910 cm^-1^. In addition, the absorption band of linoleic acid is 5952–6757 cm^-1^ [24], which is largely derived from fatty acids in flesh. Since the absorption band of monounsaturated fatty acids is 5662 cm^-1^ [25], this is also consistent with the flesh spectra at 5662 cm^-1^. In addition, it was also found that light, drying temperature and soaking dry-promoting agent solution during the production process effects on the content of coutaric and fertaric acids, quercetin-3-O-glucoside and rutin in raisins, in existing research, the traditional method favors the formation of coumaric and ferric acids, quercetin-3-O-glucoside and rutin in raisins at 60 ℃ [26–28]. These results largely explain the reason why the peaks of the three types of flesh spectra in Fig 3(B) are significantly different at 5650, 5910, 6400 and 6820 cm^-1^. Therefore, the differences in the content of the above substances between skin and flesh led to better discrimination in the average spectra of skin about 4000–5000 cm^-1^ and flesh about 5500–7000 cm^-1^. This provides a theoretical basis for feature fusion of skin and flesh spectra to improve the model’s classification performance.

### 3.2 Dataset division and model evaluation

In the experiment, the samples of training set and test set were randomly divided according a ratio of 7:3. In feature extraction, the cumulative contribution rate of skin and flesh spectra was more than 99%. Feature fusion was to combine the features of skin and flesh into a new feature matrix. After fusion, the samples corresponding to the training set and the test set remained unchanged.

In multi-classification problems, precision, recall, accuracy and F score are often used as indicators for model evaluation. In this experiment, we calculated the above evaluation indexes through the confusion matrix, as shown in S1 Table. In model comparison, this study mainly refer to Macro-F1 score and accuracy, and their calculation formulas as follows [29]:

$$\mathrm{Precision}=\frac{TP}{TP+FP}$$ (1)

Recall=TPTP+FN (2)

F1score=2.Precision.RecallPrecision+Recall (3)

$$\mathrm{Macro}-F1\;\mathrm{score}=\frac{1}{n}\overset{n}{\underset{i=1}{\sum }}F_{1i}\;\mathrm{score}$$ (4)

$$\mathrm{Accuracy}=\frac{TP+TN}{TP+FN+FP+TN}$$ (5)

TP is true positive, which represents the target samples correctly classified by the model; FN is false negative, which means that the target samples are incorrectly classified as a nontarget sample by the model; FP is false positive, which means that the sample that does not belong to the predetermined target is incorrectly predicted as the target sample by the model; FP is false positive, which means that those samples that do not belong to the predetermined target are incorrectly predicted by the model as target samples; TN is true negative, which means that nontarget samples are correctly classified by the model as samples that do not belong to the predetermined target. *F*_1*i*_ *score* represents the *F*1 score of each class of sample; *Macro*−*F*1 score is the average value of the *F*1 score.

In the experiment, n = 3, n means the number of sample classes in a multi-class problem.

### 3.3 Linear model

KNN is a linear classification model. Determine the category of a sample to be classified according to the category of the nearest few samples. KNN has the advantages of insensitivity to abnormal samples and fast training speed. In addition, KNN has obtained great experimental results in mushroom species identification and mature watermelon detection [30, 31]. Combined with their research, k value was set to 5.

LDA maps feature data to a new vector space. The projected samples satisfy the minimum distance between the same class of samples in the new space; the distance between samples of different classes is the largest. Therefore, LDA is widely used in the food classification and the auxiliary diagnosis of diseases [32, 33].

**Fig 4:**
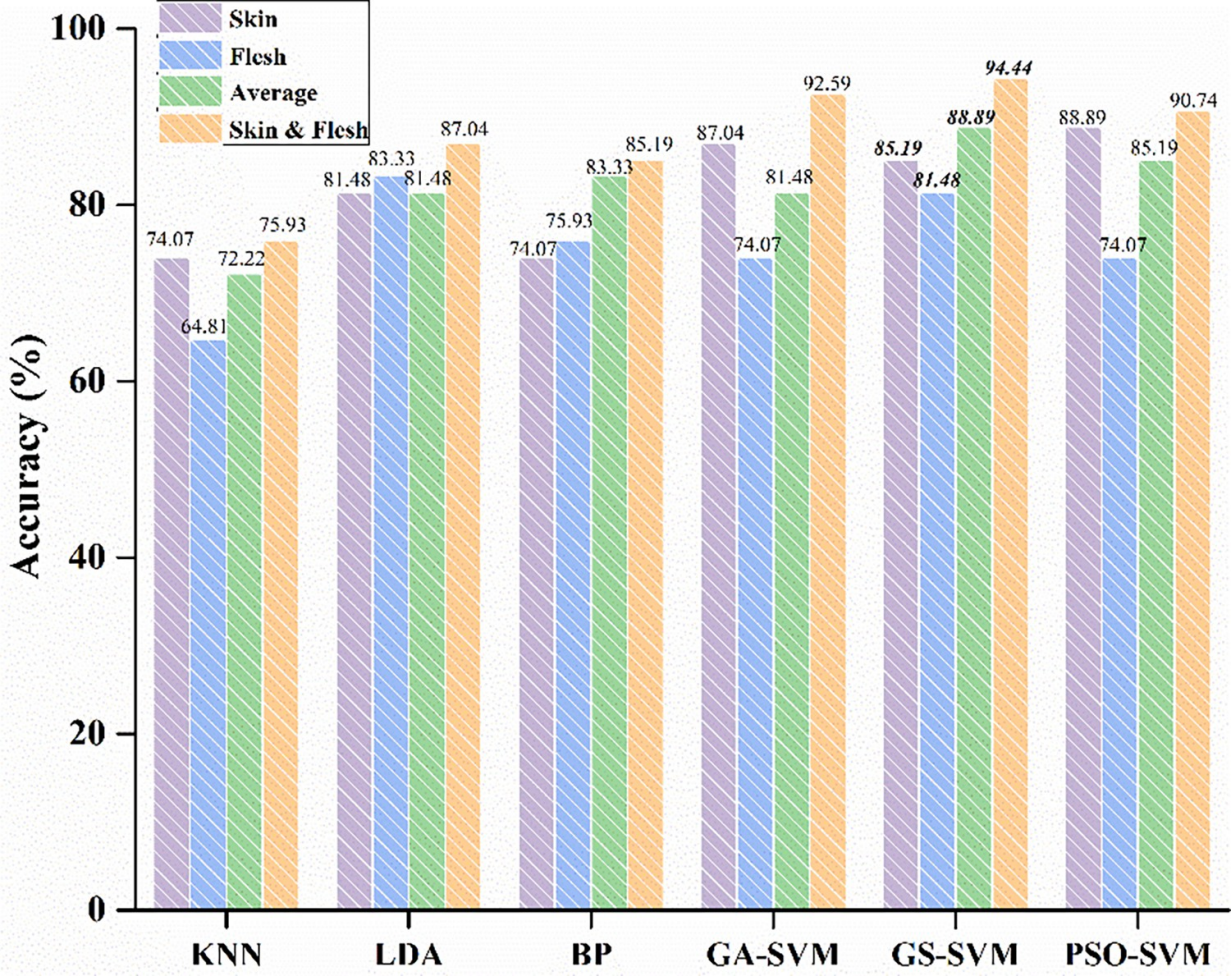
The average accuracy of the models for three types of raisins.

**Fig 5:**
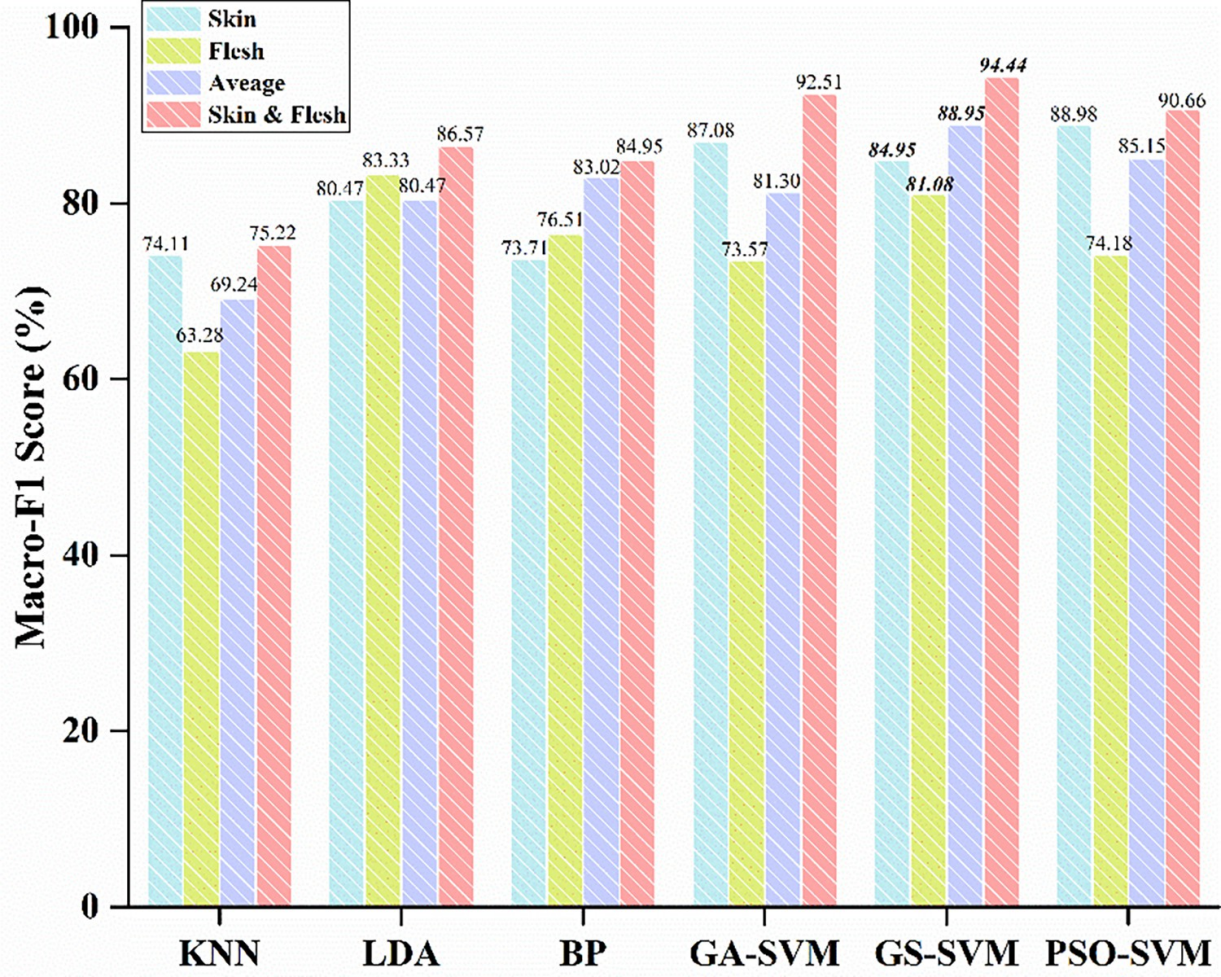
The Macro-F1 score of the models.

As shown in Figs 4 and 5, the classification accuracy and Macro-F1 score of KNN are 74.07% and 74.11%, respectively, only using spectral features of skin; the classification accuracy and Macro-F1 score are 64.81% and 63.28%, respectively, only using the spectral features of flesh; after the spectra averaged, the classification accuracy and Macro-F1 score of KNN are 72.22% and 69.24%, respectively. With feature fusion, the classification accuracy and Macro-F1 score are 75.93% and 75.22%, respectively.

In the LDA model, the classification accuracy and Macro-F1 score are 81.48% and 80.47%, respectively, using the spectral features of skin; the classification accuracy and Macro-F1 score are both 83.33% with the spectral features of flesh; after spectra averaged, the classification accuracy and Macro-F1 score of LDA are 81.48% and 80.47%, respectively. With feature fusion, the classification accuracy and Macro-F1 score are 87.04% and 86.57%.

### 3.4 Nonlinear model

BP is a feedforward neural network, its network structure includes an input layer, hidden layer and output layer. Through the errors back propagation, the weights and biases from the hidden layer to the output layer and the input layer to the hidden layer are adjusted respectively. BP not only has strong adaptability and generalization ability but also has certain advantage in solving nonlinear problems [34]. In the experiment, the iterative period of training was set to 500; The learning rate was set to 0.001.

SVM is a classical nonlinear classification model. It has strong advantages in solving the problems of fewer training samples and more categories of sample [35]. In addition, penalty coefficient C and kernel function g play a key role in finding the optimal decision plane [36, 37]. In the experiment, particle swarm optimization (PSO), genetic algorithm (GA) and grid search (GS) algorithm were used to optimize the value of parameters C and g. The models used 10-fold cross-validation and Gaussian kernel was selected as the kernel function. In the PSO algorithm, the initial local search capability was set to 1.5; the global search capability was set to 1.7; the maximum population number was set to 20; the maximum evolution number was set to 200; and the ranges of C and g were within [10^–1^, 10^2^] and [10^–2^, 10^3^], respectively. In the GA algorithm, the ranges of C and g were within (0, 10^2^] and [0, 10^3^], respectively; the population number was set to 20; and the maximum evolutionary quantity was set to 200. In the GS algorithm, the C and g parameter optimization ranges were within [–10, 10], and the step size was set to 0.8.

As shown in Figs 4 and 5, the classification accuracy and Macro-F1 score of BP are 74.07% and 73.71%, respectively, using the spectral features of skin; the classification accuracy and Macro-F1 score are 75.93% and 76.51%, respectively, with the spectral features of flesh; after the two spectra averaged, the classification accuracy and Macro-F1 score of the model are 83.33% and 83.02%, respectively. With feature fusion, the classification accuracy and Macro-F1 score are 85.19% and 84.95%.

With the spectral features of skin, the classification accuracies of GA-SVM, GS-SVM and PSO-SVM are 87.04%, 85.19% and 88.89%, respectively, and the Macro-F1 scores are 87.08%, 84.95% and 88.98%, respectively; with the spectral features of flesh, the classification accuracies are 74.07%, 81.48% and 74.07%, respectively, and the Macro-F1 scores are 73.57%, 81.08% and 74.18%, respectively; with spectra averaging, the classification accuracies of GA-SVM, GS-SVM and PSO-SVM are 81.48%, 88.89% and 85.19%, respectively, and the Macro-F1 scores are 81.30%, 88.95% and 85.15%, respectively; After feature fusion, the classification accuracies of GA-SVM, GS-SVM and PSO-SVM are 92.59%, 94.44% and 90.74%, respectively, and the Macro-F1 scores are 92.51%, 94.44% and 90.66%, respectively.

### 3.5 Results comparison

S1 Table shows that PSO-SVM has the highest classification accuracy of 88.89% by using skin features and the model also has the highest Macro-F1 score of 88.98%; LDA has the best classification accuracy and Macro-F1 score of 83.33% by using flesh features; with the average processing of skin and flesh spectra, GS-SVM with the highest accuracy and Maro-F1 score are 88.89% and 88.95%, respectively. After feature fusion, GS-SVM with the highest classification accuracy and Maro-F1 score are both of 94.44%. In addition, in the linear classification model, LDA’s classification accuracy and Macro-F1 score are higher than KNN regardless of single-class features or feature fusion; in the nonlinear classification model, the classification accuracy and Macro-F1 score of SVM after feature fusion are higher than BP and the performance of GS-SVM model is the best. As shown in Table 3, compared with averaging the spectra of the two, the classification accuracy and Macro-F1 score of KNN improved by 3.7% and 5.98%, respectively, after spectral features fusion. Besides, the classification accuracy and Macro-F1 score of LDA improved by 5.56% and 6.10%, respectively; the BP improved by 1.86% and 1.93%, respectively; the GA-SVM improved by 11.11% and 11.22%, respectively; the GS-SVM improved by 5.56% and 5.50%, respectively; and the PSO-SVM improved by 5.56% and 5.51%, respectively.

**Table 3:** Compared with other methods, the improvement of model with feature fusion.

Improvement	LDA & Skin	LDA & Flesh	LDA & Average
Macro-F1(%)	6.10	3.24	6.10
Accuracy (%)	5.56	3.70	5.56
Improvement	KNN & Skin	KNN & Flesh	KNN & Average
Macro-F1(%)	1.11	11.93	5.97
Accuracy (%)	1.85	11.11	3.70
Improvement	BP & Skin	BP & Flesh	BP & Average
Macro-F1(%)	11.24	8.44	1.93
Accuracy (%)	11.11	9.26	1.86
Improvement	PSO-SVM & Skin	PSO-SVM & Flesh	PSO-SVM & Average
Macro-F1(%)	1.67	16.47	5.51
Accuracy (%)	1.85	16.67	5.56
Improvement	GS-SVM & Skin	GS-SVM & Flesh	GS-SVM & Average
Macro-F1(%)	9.49	13.36	5.49
Accuracy (%)	9.26	12.96	5.56
Improvement	GA-SVM & Skin	GA-SVM & Flesh	GA-SVM & Average
Macro-F1(%)	5.43	18.94	11.22
Accuracy (%)	5.56	18.52	11.11

## 4. Discussion

In the experiment, the spectrum shows that the differences of characteristic peaks of lipids and phenols (4330, 4400 cm^-1^) in skin spectra of the different varieties of raisins were more obvious than that of flesh spectra; the differences of characteristic peaks of fatty acids, amino acids and carbohydrates (5650, 5910 cm^-1^) in flesh spectra were more obvious than that of skin spectra. Studies have shown that skin and flesh are more susceptible to climate and content of water in the external environment, and only recording spectral data on the skin will limit the model classification performance [11]. Based on above reasons, this study recorded the spectral data of raisins on the skin and flesh, and compared the results of the experiments using only the skin spectra, only the flesh spectra, the average spectra of the them and feature fusion of them.

In addition, since the recorded spectra is subject to stray light in the instrument, ambient temperature and the non-linear response of the detector, these will lead to poor classification of the linear model [38, 39]. Experiments show that the feature fusion method can not only effectively overcome the influence of these factors, but also improve the classification accuracy, recall rate and F1 score of the linear model. Compared with the linear model, although the nonlinear response has less impact on the nonlinear model, the way of feature fusion can also further improve the classification performance of the nonlinear model.

The results showed that after ten-fold cross-validation of the PCA-GS-SVM model with spectral feature fusion, the highest classification accuracy and Macro-F1 score were both 94.44%. Compared with the results of spectra average processed, the classification accuracy was improved by 7.4%. It can be concluded that the high performance of SVM in classification accuracy and Macro-F1 score may be derived from it as a convex optimization problem, so it can achieve better results on small batch samples problems [40], or may be related to the selected Gaussian kernel function. In contrast, the linear classification models are easily affected by the nonlinear response in the spectral data, which may be an important reason for limiting the performance of the linear models. It was also found that the feature fusion approach could overcome the effect of nonlinear response in the spectral data to some extent.

As shown in Fig 3, in the range of 4000–5000 cm^-1^, the differences of skin spectra of the three kinds of raisins were more obvious than that of flesh; in the range of 5500–7000 cm^-1^, the differences of flesh spectra were more obvious than skin. This phenomenon may be caused by factors such as climate and content of water in the origins of raisins, or may be related to the type of drying accelerator used and drying temperature. The average spectra of skin and meat (Fig 3(C)) show that the difference in spectral characteristic peaks decreases at 4330, 4400, 4758 and 5120 cm^-1^. This also shows that the fused feature matrix has more obvious difference between the skin and flesh spectra, which may be the reason for the improvement of model classification performance.

## 5. Conclusion

This experiment used NIR spectra combined with machine learning algorithms to quickly classify the raisins of Hongxiangfei, Manaiti and Munage. This study compared the experimental results of using only the skin spectra, only the flesh spectra, the average spectra of the them and feature fusion of them. The experimental results show that the accuracy of spectral feature fusion was higher than other three experiments regardless of whether using linear classification models or nonlinear classification models. This may provide a new strategy of spectra recording and feature fusion for the future food field.

## Supporting information

S1 Table The precision, recall and f1score of the models. (PDF)

S1 File Spectral data. (ZIP)
