# Supplementary material for: Identification of multiple raisins by feature fusion combined with NIR spectroscopy
Source: PLoS One. 2022 Jul 14;17(7):e0268979. doi: 10.1371/journal.pone.0268979 (PMC9282468; doi:10.1371/journal.pone.0268979)
Supplement: S1 Table — (PDF) [file pone.0268979.s001.pdf]

Table S1 The precision, recall and f1score of the models

| | LDA & Skin | | | LDA & Flesh | | |
|---|---|---|---|---|---|---|
| | HXF (%) | MNT (%) | MNG (%) | HXF (%) | MNT (%) | MNG (%) |
| Precision (%) | 90.00 | 72.73 | 83.33 | 100.00 | 80.95 | 75.00 |
| Recall (%) | 100.00 | 88.89 | 55.56 | 72.22 | 94.44 | 83.33 |
| F1score (%) | 94.74 | 80.00 | 66.67 | 83.87 | 87.18 | 78.95 |
| | LDA & Average | | | LDA & Fusion | | |
| | HXF (%) | MNT (%) | MNG (%) | HXF (%) | MNT (%) | MNG (%) |
| Precision (%) | 90.00 | 72.73 | 83.33 | 94.74 | 77.27 | 92.31 |
| Recall (%) | 100.00 | 88.89 | 55.56 | 100.00 | 94.44 | 66.67 |
| F1score (%) | 94.74 | 80.00 | 66.67 | 97.30 | 85.00 | 77.42 |
| | KNN & Skin | | | KNN & Flesh | | |
| | HXF (%) | MNT (%) | MNG (%) | HXF (%) | MNT (%) | MNG (%) |
| Precision (%) | 88.89 | 63.16 | 70.59 | 65.00 | 62.50 | 70.00 |
| Recall (%) | 88.89 | 66.67 | 66.67 | 72.22 | 83.33 | 38.89 |
| F1score (%) | 88.89 | 64.86 | 68.57 | 68.42 | 71.43 | 50.00 |
| | KNN & Average | | | KNN & Fusion | | |
| | HXF (%) | MNT (%) | MNG (%) | HXF (%) | MNT (%) | MNG (%) |
| Precision (%) | 80.00 | 68.00 | 66.67 | 80.00 | 71.43 | 76.92 |
| Recall (%) | 88.89 | 94.44 | 33.33 | 88.89 | 83.33 | 55.56 |
| F1score (%) | 84.21 | 79.07 | 44.44 | 84.21 | 76.92 | 64.52 |
| | BP & Skin | | | BP & Flesh | | |
| | HXF (%) | MNT (%) | MNG (%) | HXF (%) | MNT (%) | MNG (%) |
| Precision (%) | 80.00 | 68.75 | 72.22 | 91.67 | 58.62 | 100.00 |
| Recall (%) | 88.89 | 61.11 | 72.22 | 61.11 | 94.44 | 72.22 |
| F1score (%) | 84.21 | 64.71 | 72.22 | 73.33 | 72.34 | 83.87 |
| | BP & Average | | | BP & Fusion | | |
| | HXF (%) | MNT (%) | MNG (%) | HXF (%) | MNT (%) | MNG (%) |
| Precision (%) | 84.62 | 70.83 | 100.00 | 80.95 | 86.67 | 88.89 |
| Recall (%) | 61.11 | 94.44 | 94.44 | 94.44 | 72.22 | 88.89 |
| F1score (%) | 70.97 | 80.95 | 97.14 | 87.18 | 78.79 | 88.89 |
| | PSO-SVM & Skin | | | PSO-SVM & Flesh | | |
| | HXF (%) | MNT (%) | MNG (%) | HXF (%) | MNT (%) | MNG (%) |
| Precision (%) | 93.75 | 100.00 | 78.26 | 75.00 | 86.67 | 65.22 |
| Recall (%) | 83.33 | 83.33 | 100.00 | 66.67 | 72.22 | 83.33 |
| F1score (%) | 88.24 | 90.91 | 87.80 | 70.59 | 78.79 | 73.17 |
| | PSO-SVM & Average | | | PSO-SVM & Fusion | | |
| | HXF (%) | MNT (%) | MNG (%) | HXF (%) | MNT (%) | MNG (%) |
| Precision (%) | 87.50 | 88.89 | 80.00 | 94.12 | 93.75 | 85.71 |
| Recall (%) | 77.78 | 88.89 | 88.89 | 88.89 | 83.33 | 100.00 |
| F1score (%) | 82.35 | 88.89 | 84.21 | 91.43 | 88.24 | 92.31 |
| | GS-SVM & Skin | | | GS-SVM & Flesh | | |

| | HXF (%) | MNT (%) | MNG (%) | HXF (%) | MNT (%) | MNG (%) |
|---|---|---|---|---|---|---|
| Precision (%) | 94.74 | 78.95 | 81.25 | 80.95 | 83.33 | 80.00 |
| Recall (%) | 100.00 | 83.33 | 72.22 | 94.44 | 83.33 | 66.67 |
| F1score (%) | 97.30 | 81.08 | 76.47 | 87.18 | 83.33 | 72.73 |
| | GS-SVM & Average | | | GS-SVM & Fusion | | |
| | HXF (%) | MNT (%) | MNG (%) | HXF (%) | MNT (%) | MNG (%) |
| Precision (%) | 94.12 | 80.95 | 93.75 | 100.00 | 89.47 | 94.12 |
| Recall (%) | 88.89 | 94.44 | 83.33 | 100.00 | 94.44 | 88.89 |
| F1score (%) | 91.43 | 87.18 | 88.24 | 100.00 | 91.89 | 91.43 |
| | GA-SVM & Skin | | | GA-SVM & Flesh | | |
| | HXF (%) | MNT (%) | MNG (%) | HXF (%) | MNT (%) | MNG (%) |
| Precision (%) | 94.12 | 83.33 | 84.21 | 93.75 | 64.00 | 69.23 |
| Recall (%) | 88.89 | 83.33 | 88.89 | 83.33 | 88.89 | 50.00 |
| F1score (%) | 91.43 | 83.33 | 86.49 | 88.24 | 74.42 | 58.06 |
| | GA-SVM & Average | | | GA-SVM & Fusion | | |
| | HXF (%) | MNT (%) | MNG (%) | HXF (%) | MNT (%) | MNG (%) |
| Precision (%) | 93.75 | 73.91 | 80.00 | 90.00 | 89.47 | 100.00 |
| Recall (%) | 83.33 | 94.44 | 66.67 | 100.00 | 94.44 | 83.33 |
| F1score (%) | 88.24 | 82.93 | 72.73 | 94.74 | 91.89 | 90.91 |

HXF is the Hongxiangfei; MNT is the Manaiti; MNG is the Munage.
